# Optimization of chemical and enzymatic hydrolysis for production of chicken blood protein hydrolysate rich in angiotensin-I converting enzyme inhibitory and antioxidant activity

**DOI:** 10.1016/j.psj.2021.101047

**Published:** 2021-02-13

**Authors:** R. Nikhita, N.M. Sachindra

**Affiliations:** ∗ Department of Meat and Marine Sciences, CSIR- Central Food Technological Research Institute (CFTRI), Mysore, India 570020; † Academy of Scientific and Innovative Research (AcSIR), Ghaziabad, India 201002

**Keywords:** chicken red blood cell, alcalase, degree of hydrolysis, DPPH scavenging, ACE inhibition

## Abstract

Response surface methodology was adopted to optimize hydrolysis conditions for the production of antioxidant and angiotensin-I converting enzyme (**ACE**) inhibitory peptides from chicken red blood cells by both enzymatic and acid hydrolysis. During acid hydrolysis, temperature (*P* < 0.001) and acid concentration (*P* < 0.001) influenced the degree of hydrolysis (**DH%**) and 1,1-diphenyl-2-picrylhydrazyl (**DPPH**) radical scavenging activity of the hydrolysate while ACE inhibitory activity of the hydrolysate was strongly influenced by acid concentration (*P* < 0.001). Temperature and time of hydrolysis had no effect (*P* > 0.05) on the ACE inhibitory activity of the hydrolysate. Acid hydrolysis conditions of 50°C, 32 h, and 0.03 N hydrochloric acid resulted in optimum DH% (33.1%), optimum DPPH scavenging activity (46%), and optimum ACE inhibitory activity (43.7%) of the hydrolysate. During enzymatic hydrolysis of chicken red blood cells, DH% was influenced by the temperature of hydrolysis (*P* < 0.001) and enzyme concentration (*P* < 0.001). DPPH scavenging of the hydrolysate was marginally (*P* < 0.05) influenced by the temperature of hydrolysis and ACE inhibitory activity of the hydrolysate was highly influenced by temperature (*P* < 0.001) and enzyme concentration (*P* < 0.001). Enzyme hydrolysis conditions of 60°C, 150 min, and 2.5% alcalase resulted in maximum DH% of 63.9%, while the highest DPPH scavenging activity (75%) of hydrolysate was observed under the hydrolysis conditions of 60°C, 30 min, and 2.5% of the enzyme. Optimum ACE inhibitory activity (45%) of the hydrolysate was achieved at hydrolysis conditions of 2.5% alcalase, 120 min of hydrolysis at 60°C. ACE inhibitory activity of the enzymatically hydrolyzed product was directly proportional to DH%, while DPPH activity was inversely proportional to DH%. DPPH scavenging activity of the acid hydrolysate was recorded at a lower range (34.8–56.9%) compared to the enzyme hydrolysate (40.4–77.4%), while ACE inhibitory activity of both the hydrolysates was observed in the same range (18.7–49.4 and 14.2–47.7% for acid and enzyme hydrolysate, respectively). This study indicated that chicken red blood cells could be successfully hydrolyzed by both chemical and enzymatic methods to obtain hydrolysates having antioxidant and ACE inhibitory activity.

## Introduction

Animal blood is the main by-product of meat processing derived from the activity of industrial slaughterhouses. Blood corpuscle constitutes 40% of blood, accounts for more than 50% of the total protein content, and is a potential source of protein used as food and feed ingredient (Zheng et al., 2014). Blood is a red fluid made up of water, cells, enzymes, proteins, and other organic and inorganic substances that can be separated into 2 fractions, the cellular fraction and plasma. The cellular fraction corresponds to 30 to 40% of blood and is dispersed within the liquid fraction, which is known as the plasma (which comprises up to 60%) (Bah et al., 2013).

Animal blood is mainly used for the production of plasma, which is used in meat and other food products due to better functional properties like emulsification and heat coagulation properties (Ramos-Clamont et al., 2006; Davila et al., 2007). Plasma is separated from raw blood by centrifugation, and the red cell fraction, even though high in protein, is rarely used in food due to its dark color and blood flavor (Gomez-Juarez et al., 1999a). The utilization of blood cells in food requires decolorization or alternative techniques to mask the heme pigment in processing (Wismer-Perdesen, 1998). Enzymatic hydrolysis of hemoglobin, wherein the heme pigment is separated from globin, has been suggested as a method of decolorization, and the resultant hydrolysate was found to have good functional properties (Clark et al., 1987; Yang and Lin, 1998; Gomez-Juarez et al., 1999b).

Several studies have been conducted on the preparation of protein hydrolysates from animal blood. However, studies on the preparation of protein hydrolysate from chicken blood are scarce. Marquez and Vazquez (1999) prepared bovine hemoglobin hydrolysate using alcalase. Perez-Galvez et al. (2011) adopted bi-objective optimization of the enzymatic hydrolysis of porcine blood protein for use as liquid fertilizer. Belhocine et al. (2000) developed a theoretical kinetic model based on the Michaelis–Menten relation and the substrate mass balance for successful enzymatic hydrolysis of hemoglobin in a continuous membrane bioreactor. Konieczny et al. (2005) developed a hydrolysate with good functional properties from porcine red blood cells. Seo et al. (2015) optimized the enzyme hydrolysis of bovine plasma by alcalase using response surface methodology (**RSM**) with respect to the degree of hydrolysis (**DH%**) and antioxidant activity of the hydrolysate. Use of neutral protease for hydrolysis in porcine blood cells has been reported by Zheng et al. (2013).

Protein hydrolysates are known to possess high nutritional value and also demonstrate functional activities like antioxidant activity and angiotensin-I converting enzyme (**ACE**) inhibitory activity (Amarowicz and Shahidi, 1997; Hyun and Shin, 2000; Kong and Xiong, 2006). Yu et al. (2006) isolated ACE inhibitory peptides by peptic digestion of porcine hemoglobin. Enzymatic hydrolysis of animal blood plasmas, using fungal protease preparations, was found to produces hydrolysates with high antioxidant properties (Bah et al., 2015). Verma et al. (2017) demonstrated the antioxidant and antimicrobial activity of porcine blood hydrolysate prepared by using trypsin and alcalase. Yang et al. (2020) optimized the enzymatic hydrolysis conditions for preparation of hydrolysate from duck blood plasma, which showed 64.84% 1,1-diphenyl-2-picrylhydrazyl (**DPPH**) radical scavenging activity. Jin et al. (2020) reported that porcine blood protein hydrolysate exhibits strong antibacterial activity against *Bacillus cereus*. Chicken blood has been used as a raw material for the preparation of antioxidant peptides (Zheng et al., 2018). Wongngam et al. (2020) obtained chicken blood hydrolysate with 37.7% ACE inhibitory activity using alcalase for hydrolysis. Furthermore, they also demonstrated the in vivo antihypertensive activity of blood hydrolysate using a rat model.

Response surface methodology, a mathematical and statistical technique, is an effective method to understand the effect of different process variables and their interaction on the outcome of the process and to determine the optimum conditions for maximizing the output of the process (Zheng et al., 2014). The present investigation aimed to determine the influence of the hydrolysis conditions during the preparation of chicken blood protein hydrolysate by both enzymatic and acid hydrolysis methods. Optimization of the hydrolysis condition was carried out with respect to DH%, antioxidant activity (DPPH scavenging), and ACE inhibitory activity of protein hydrolysate.

## Materials and methods

### Chicken Blood

Chicken blood was hygienically collected directly into sterilized glass bottles using sodium citrate solution (3.8% w/v) as an anticoagulant (1:0.35 was the blood:anticoagulant solution) after slaughter of bird by neck cutting, and transported to the laboratory in ice box. The whole blood was centrifuged for 15 min at 2,500 × *g* to obtain red blood cells. The red blood cells were diluted to a 1:1 ratio using distilled water and freeze-dried. The chemicals used were of analytical grade and were from Sigma-Aldrich (Mumbai, India).

### Experimental Design

Three-level full factorial designs with 3 independent variables, namely temperature (X1), time (X2), and concentration (X3), were applied to optimize the condition for hydrolyzing chicken red blood cells. The design included 3 independent combinations and 3 replicates at the center of the experimental design, chosen to maximize the predictive capacity of the models. Also, the experimental points were generated on a sphere around the center point to ensure that the variation of the model prediction is constant for all points equidistant from the center. The ranges for independent variables were selected based on preliminary experiments. Independent factors and their coded and actual levels used in RSM studies for optimizing hydrolysis conditions using acid and enzyme are provided in Table 1. The experimental design of 27 combinations used for acid and enzyme hydrolysis is shown in Tables 2 and 3.

**Table 1:** Independent factors and their coded and actual levels used in RSM studies for optimizing hydrolysis conditions using (i) acid and (ii) enzyme.

Factor	Levels		
	−1	0	+1
X1, temperature (°C)	30	50	70
X2, time (h)	16	32	48
X3, acid concentration (N)	0.01	0.03	0.05

Factor	Levels		
	−1	0	+1
X1, temperature (°C)	20	40	60
X2, time (min)	30	90	150
X3, enzyme concentration (% w/v)	0.5	1.5	2.5

Abbreviation: RSM, response surface methodology.

**Table 2:** Experimental design for acid hydrolysis of chicken red blood cells.

Run number	Temperature, X1 (°C)	Incubation time, X2 (hour)	Acid concentration, X3 (% w/v)	Degree of hydrolysis (%)		DPPH scavenging (%)		ACE inhibition (%)	
				Observed	Predicted	Observed	Predicted	Observed	Predicted
1	30	16	0.01	4.49	3.54	43.62	41.37	18.72	22.54
2	30	16	0.03	11.39	11.69	48.30	47.50	48.04	44.43
3	30	16	0.05	16.94	13.31	54.14	53.19	39.39	44.28
4	30	32	0.01	11.39	7.48	37.98	39.95	22.91	22.41
5	30	32	0.03	17.62	16.28	44.99	46.68	46.37	44.02
6	30	32	0.05	12.01	18.54	53.40	52.98	43.58	43.59
7	30	48	0.01	8.65	6.59	42.25	41.52	21.79	23.71
8	30	48	0.03	10.83	16.03	46.26	48.86	49.44	45.04
9	30	48	0.05	19.10	18.94	56.88	55.76	44.13	44.33
10	50	16	0.01	11.24	17.45	42.75	43.39	25.70	24.81
11	50	16	0.03	28.21	27.98	47.53	48.19	48.32	43.65
12	50	16	0.05	30.60	31.97	49.46	52.54	43.02	40.45
13	50	32	0.01	13.63	21.96	39.44	40.59	24.58	25.21
14	50	32	0.03	40.21	33.14	47.73	45.99	43.30	43.77
15	50	32	0.05	39.22	37.78	49.64	50.95	39.94	40.30
16	50	48	0.01	17.12	21.65	42.25	40.78	25.42	27.04
17	50	48	0.03	44.97	33.47	49.32	46.79	44.13	45.32
18	50	48	0.05	38.96	38.76	53.45	52.35	37.71	41.57
19	70	16	0.01	3.35	−3.15	39.88	40.03	27.09	25.15
20	70	16	0.03	6.03	9.76	42.85	43.49	33.80	40.94
21	70	16	0.05	16.44	16.13	47.69	46.51	36.87	34.69
22	70	32	0.01	6.29	1.95	35.13	35.85	28.49	26.09
23	70	32	0.03	9.87	15.50	42.67	39.91	36.87	41.60
24	70	32	0.05	24.91	22.52	45.45	43.54	36.03	35.07
25	70	48	0.01	3.52	2.21	34.83	34.66	30.73	28.46
26	70	48	0.03	11.12	16.41	37.10	39.33	42.18	43.69
27	70	48	0.05	23.87	24.07	41.28	43.57	40.50	36.88
*R*^2^				0.8515		0.9177		0.8892	
Correlation coefficient (*r*) with DH						0.5510		0.4812	

Abbreviations: ACE, angiotensin-I converting enzyme; DH, degree of hydrolysis; DPPH, 1,1-diphenyl-2-picrylhydrazyl.

**Table 3:** Experimental design for preparation of blood protein hydrolysates by enzyme hydrolysis.

Run number	Temperature, X1 (°C)	Incubation time, X2 (min)	Enzyme concentration, X3 (% w/v)	Degree of hydrolysis (%)		DPPH scavenging (%)		ACE inhibition (%)	
				Observed	Predicted	Observed	Predicted	Observed	Predicted
1	20	30	0.5	28.42	27.03	40.44	40.53	14.25	17.06
2	20	30	1.5	30.67	30.11	55.84	54.33	18.72	20.58
3	20	30	2.5	34.85	34.21	70.76	64.98	25.42	21.81
4	20	90	0.5	25.37	25.76	53.13	46.13	18.72	19.41
5	20	90	1.5	29.81	28.30	55.53	56.31	22.63	22.98
6	20	90	2.5	32.89	31.85	59.54	63.33	28.77	24.25
7	20	150	0.5	23.74	26.10	52.02	55.20	18.44	19.56
8	20	150	1.5	24.75	28.10	58.53	61.75	24.58	23.17
9	20	150	2.5	32.06	31.11	61.94	65.15	21.79	24.49
10	40	30	0.5	31.30	33.62	57.42	56.95	22.07	19.30
11	40	30	1.5	33.83	36.34	63.60	66.38	26.54	26.36
12	40	30	2.5	35.82	40.07	62.34	72.66	27.93	31.12
13	40	90	0.5	37.35	34.74	40.85	57.56	25.70	21.77
14	40	90	1.5	39.80	36.91	66.80	63.36	27.65	28.87
15	40	90	2.5	38.04	40.10	70.89	66.02	28.77	33.69
16	40	150	0.5	39.43	37.47	69.58	61.63	26.26	22.03
17	40	150	1.5	42.20	39.10	69.82	63.82	28.49	29.18
18	40	150	2.5	42.32	41.74	69.92	62.85	32.96	34.04
19	60	30	0.5	52.89	51.68	67.72	68.21	24.58	23.62
20	60	30	1.5	55.29	54.03	77.41	73.27	35.20	34.22
21	60	30	2.5	61.40	57.39	76.97	75.19	41.90	42.52
22	60	90	0.5	51.76	55.19	70.07	63.82	22.91	26.21
23	60	90	1.5	55.26	56.99	64.25	65.27	36.31	36.85
24	60	90	2.5	59.37	59.81	64.31	63.56	47.77	45.20
25	60	150	0.5	61.63	60.31	61.72	62.91	22.63	26.59
26	60	150	1.5	59.84	61.57	53.44	60.73	39.39	37.28
27	60	150	2.5	63.37	63.84	52.48	55.40	47.49	45.67
*R*^2^				0.9705		0.6386		0.9059	
Correlation coefficient (*r*) with DH						0.3839		0.7585	

Abbreviations: ACE, angiotensin-I converting enzyme; DH, degree of hydrolysis; DPPH, 1,1-diphenyl-2-picrylhydrazyl.

### Hydrolysis of Red Blood Cells

#### Acid Hydrolysis

Hydrochloric acid (**HCl**) was added to 5% red blood cells in different concentrations, and hydrolysis was carried out at 30°C, 50°C, and 70°C. The samples were withdrawn at 16, 32, and 48 h in aliquots for analysis. Hydrolysis was terminated by neutralizing the hydrolysates using 1 N sodium hydroxide. The hydrolysate was centrifuged at 8,000 × *g* for 20 min to remove the insoluble material. The aliquots were used to study the DH%, DPPH radical scavenging activity, and ACE inhibitory activity.

#### Enzymatic Hydrolysis

The enzyme alcalase was added to 5% red blood cells in different concentrations, and hydrolysis was carried out at 20°C, 40°C, and 60°C. The samples were withdrawn at 30, 90, and 150 min in aliquots for analysis. The activity of the enzyme was terminated by heating the hydrolysate in a boiling water bath for 15 min and then cooling in ice for 5 min. The hydrolysate was centrifuged at 8,000 × *g* for 20 min to remove the insoluble material. The enzymatically hydrolyzed aliquots were used to study the DH%, DPPH radical scavenging activity, and ACE inhibitory activity.

### Determination of DH%, DPPH Scavenging Activity, and ACE Inhibitory Activity

#### Determination of α-Amino Acid and DH%

Degree of hydrolysis was measured by the method described by Thiansilakul et al. (2007). After the hydrolysis of red blood cells, trichloroacetic acid (1:1 ratio) was added to the hydrolysate and the reaction mixture was centrifuged at 8,000 × *g* for 30 min. The hydrolysate sample (10–50 μL) was diluted with 0.1 mL of 0.2 M phosphate buffer, pH 8.2, and 0.05 mL of 0.01% 2,4,6-trinitrobenzenesulfonic acid solution was added. The solution was mixed thoroughly and placed in a water bath at 50°C for 30 min in the dark. The reaction was terminated by adding 0.1 mL of 0.1 M sodium sulfite. The mixture was cooled at room temperature for 15 min. The absorbance was read at 420 nm using a Hitachi U-2900 spectrophotometer (Hitachi High-Tech Corporation, Tokyo, Japan), and α-amino acid was expressed in terms of L-leucine.

$$\text{DH}=\left(\text{Lt}-\text{L0}/{\text{L}}_{\text{max}}\right)\times 100$$

where Lt is the amount of α-amino acid released at time t; L0 is the amount of α-amino acid in red blood cell powder; and L_max_ is the total α-amino acid in red blood cells powder after acid hydrolysis (6 N HCl at 100°C for 24 h).

#### DPPH Radical Scavenging Activity

DPPH radical scavenging activity of the samples was measured by the method described by Duan et al. (2006). In brief, an aliquot of the sample was made up to 2 mL with methanol and mixed with 2 mL of 0.16 mmol DPPH in methanol and incubated at 37°C for 30 min in the dark. Methanol alone in the reaction mixture served as control. The sample blank was prepared by replacing the DPPH with methanol. The absorbance of the sample after incubation was measured at 517 nm using the Hitachi U-2900 spectrophotometer, and the scavenging activity was calculated as follows:

$$\text{Scavenging}\%=1-({\text{A}}_{\text{sample}}-{\text{A}}_{\text{sample blank}})/{\text{A}}_{\text{control}})\times 100$$

#### ACE Inhibitory Activity

The assay for ACE inhibitory activity was performed using the method of Liu et al. (2010) with slight modifications. ACE was prepared with sodium borate buffer (50 mmol L^−1^ containing 300 mmol L^−1^ NaCl at pH 8.3). Hippuryl-L-histidyl-L-leucine (**Hip-His-Leu**) was used as the substrate of ACE. A sample solution (10–50 μL) with 50 μL of ACE (0.25 units/2.5 mL) solution was pre-incubated at 37°C for 5 min, and the reaction commenced by the addition of 50 μL of 5 mmol L^−1^ Hip-His-Leu. After incubation at 37°C for 30 min, the reaction was terminated by adding 200 μL of 1 mol L^−1^ HCl. The reaction product, hippuric acid, was extracted with 1 mL of ethyl acetate. After centrifugation (3,500 × *g*, 5 min), 0.8 mL of the upper layer was transferred into the microcentrifuge tube and ethyl acetate was volatilized in a water bath at 90°C for 30 min. The residue of hippuric acid was dissolved in 0.8 mL of distilled water, and the absorbance was measured at 228 nm using the Hitachi U-2900 spectrophotometer. Inhibitory activity was calculated by using the absorbance of hippuric acid liberated from Hip-His-Leu by ACE.

Inhibitory activity(%)=Control-(Sample test-Sample blank)/Control×100

### Electrophoresis of Hydrolysate

Both the acid hydrolysate and enzyme hydrolysate prepared under optimum conditions were subjected to SDS-PAGE to determine the molecular distribution pattern of proteins and their fragments in the hydrolysate. Dried red blood cells powder and hydrolysate samples were dissolved in 0.02M phosphate buffer (pH 7.0) and 70 μg protein was loaded onto a polyacrylamide gel made of 5% stacking and 12% separating gels. The protein bands were stained with Coomassie Brilliant Blue R-250, distained, and the separated proteins and released fragments were visualized using the G-Box F3 gel documentation system (Syngene, Frederick, MD).

### Statistical Analysis

All the statistical analyses were carried out using the Statistica software (Statsoft Inc, 1999). The effect of each factor and their interactions on the dependent variables was assessed by the ANOVA technique. The optimization data were analyzed for the determination of regression coefficients to arrive at the regression equation. The regression model containing 10 coefficients including the linear and quadratic effect of factors and linear effect of interactions was assumed to describe relationships between response (Y) and the experimental factors (X1, X2, X3) as follows:where β_0_ is the constant coefficient, β_i_ is the linear coefficient of main factors, β_ii_ is the quadratic coefficient for main factors, and β_ij_ is the second-order interaction coefficient. The response variable was assigned at low and high of the observed values for desirability of 0 and 1, respectively, to get the overall desirability. The desirability function to obtain the optimum carotenoid yield was fitted by the least square method using the software. The three-dimensional response graph and profile for predicted values and desirability levels for factors were plotted using the Statistica software.

## Results and discussion

Hydrolysis of protein is influenced by the conditions adopted for the hydrolysis. The extent of hydrolysis (DH%) determines the biological activity of the hydrolysate. Although some studies on blood hydrolysate from porcine and bovine species can be found in the literature, no reports detailing the effect of hydrolysis conditions and the optimal conditions required to obtain hydrolysates from chicken blood are available. Hence, it is essential to evaluate the effect of different factors undermining the hydrolysis of chicken blood on the DH% and the biological activity of the resultant hydrolysate. As different independent factors of hydrolysis may have different effects, RSM was used to determine the effect of the individual parameters and their interactions of the DH%, antioxidant, and ACE inhibitory activity of the hydrolysate. Regression analysis of data was used to arrive at the regression equation to predict the dependent variables and to arrive at the optimum hydrolysis conditions.

### Acid Hydrolysis

**Figure 1:**
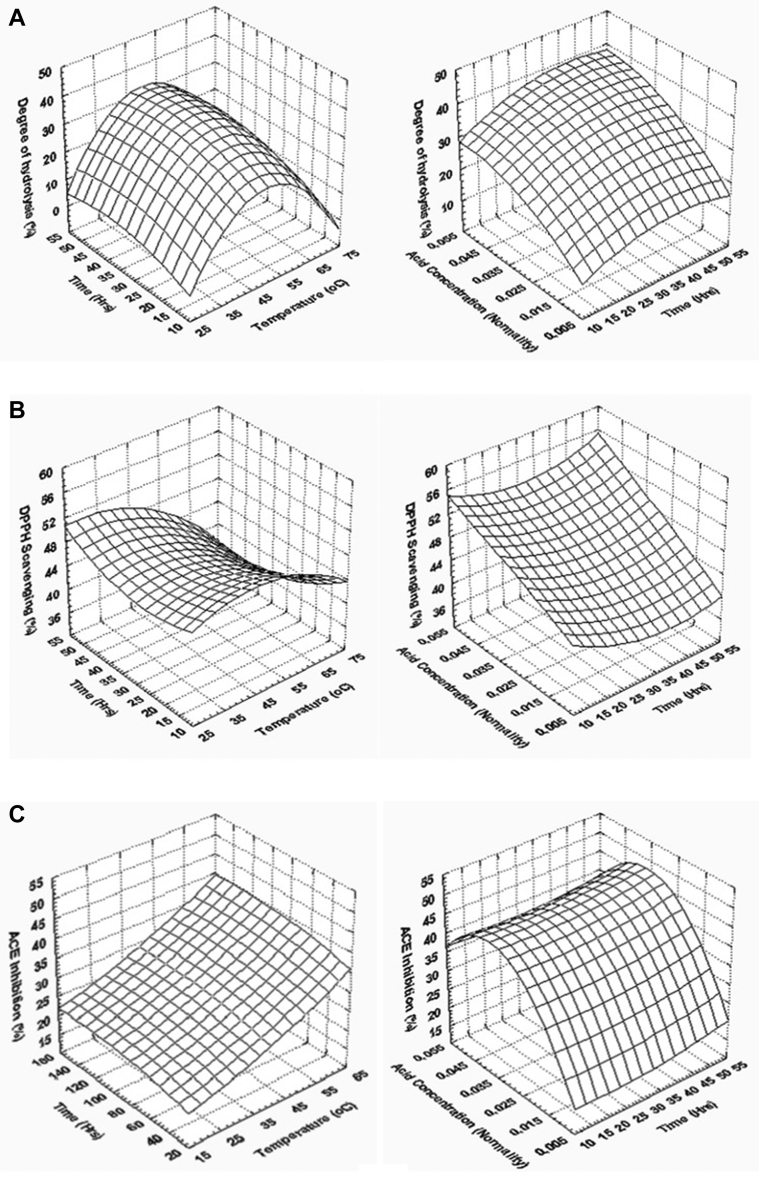
Effect of time and temperature (acid concentration = 0.03 N), time and acid concentration (temperature 50°C) on (A) degree of hydrolysis, (B) DPPH scavenging activity, and (C) ACE inhibitory activity of hydrolysate. Abbreviations: ACE, angiotensin-I converting enzyme; DPPH, 1,1-diphenyl-2-picrylhydrazyl.

Acid hydrolysis of chicken red blood cells was carried out at different combinations of acid (HCl) concentration, time, and temperature of hydrolysis to evaluate the effect of these independent factors and their interaction on DH%, DPPH scavenging, and ACE inhibitory activity of the hydrolysate. The observed values for the different independent variables are provided in Table 2. Results of the ANOVA to evaluate the effect of process variables on dependent variables are shown in Table 4. It was observed that temperature and acid concentration had an effect (*P* < 0.001) on the DH%, while the time of hydrolysis and the interaction of 3 process variables had no effect on the DH%. The response surface graphs (Figure 1A) of the effect of process variables on the DH% show that an increase in temperature of hydrolysis of up to 50°C increases the DH%, but DH% reduced when temperature increased further. There was a gradual increase in DH% with time of hydrolysis initially up to 30 h, and it stabilized thereafter. DH% increased with an increase in acid concentration.

**Table 4:** ANOVA for the effect of acid hydrolysis conditions and their interactions on degree of hydrolysis, DPPH scavenging, and ACE inhibition of protein hydrolysate.

Factor	Degree of hydrolysis				DPPH scavenging				ACE inhibition			
	Sum of squares	degrees of freedom	Mean sum of squares	F value	Sum of squares	degrees of freedom	Mean sum of squares	F Value	Sum of squares	degrees of freedom	Mean sum of squares	F value
Temperature (X1)	1,788.23	2	894.12	27.03∗∗∗	249.61	2	124.80	29.43∗∗∗	31.93	2	15.96	1.14NS
Time (X2)	170.82	2	85.41	2.58NS	22.30	2	11.15	2.63NS	15.70	2	7.85	0.56NS
Acid concentration (X3)	1,189.88	2	594.94	17.98∗∗∗	483.60	2	241.80	57.02∗∗∗	1,752.22	2	876.11	62.41∗∗∗
X1∗X2	3.99	1	3.99	0.12NS	22.77	1	22.77	5.37∗	3.44	1	3.44	0.25NS
X1∗X3	67.84	1	67.84	2.05NS	21.36	1	21.36	5.04∗	111.58	1	111.58	7.95∗
X2∗X3	5.00	1	5.00	0.15NS	4.38	1	4.38	1.03NS	0.94	1	0.94	0.07NS
Error	562.40	17	33.08		72.10	17	4.24		238.63	17	14.04	
Total SS	3,788.16	26			876.11	26			2,154.43	26		

∗∗∗*P* < 0.001; ∗*P* < 0.05.

Abbreviations: ACE, angiotensin-I converting enzyme; DPPH, 1,1-diphenyl-2-picrylhydrazyl; NS (*P* > 0.05).

DPPH radical scavenging activity of the hydrolysate was also influenced by temperature (*P* < 0.001), acid concentration (*P* < 0.001), and the interaction between temperature and time (*P* < 0.05) and acid concentration (*P* < 0.05) adopted for hydrolysis. An increase in hydrolysis temperature of up to 45°C increased the DPPH radical scavenging activity of the hydrolysate, and further increase in temperature resulted in decreased DPPH radical scavenging activity of the hydrolysate (Figure 1B). Increase in the time of hydrolysis had a marginal effect on DPPH scavenging, while the DPPH scavenging activity of the hydrolysate increased with an increase in the concentration of the acid used for hydrolysis.

ACE inhibitory activity of the hydrolysate was affected by acid concentration (*P* < 0.001) and the interaction between temperature and acid concentration (*P* < 0.001). However, temperature and time of hydrolysis had no effect (*P* > 0.05) on the ACE inhibitory activity of the hydrolysate. The response surface plots (Figure 1C) for ACE inhibitory activity of the hydrolysate show that the ACE inhibitory activity increased gradually with increase in the temperature of hydrolysis; time of hydrolysis had a marginal effect on ACE inhibition; while acid concentration of up to 0.04 N increased the ACE inhibitory activity of the hydrolysate.

Regression analysis of the experimental data was carried out to obtain the regression coefficients (Table 5) for the main effects and their interactions to fit a suitable regression equation (Eq.1) for dependent variables as a function of linear and quadratic effects of main factors and the linear-by-linear interaction effects. The regression equation was used to arrive at the predicted value of the dependent variable at each combination of the processing variables (factors). The closeness of the observed and the predicted value of the dependent variable was determined by the *R*^2^ value. High *R*^2^ values of 0.8515, 0.9177, and 0.8892 between the observed and predicted values of the DH%, DPPH radical scavenging, and ACE inhibition, respectively, indicate that the derived regression equation can be used to determine the value of the dependent variable at different levels of 3 factors, which are influencing them. However, no strong correlation was observed between DH% and DPPH radical scavenging activity (*r* = 0.55) and DH% and ACE inhibitory activity (*r* = 0.48), indicating that these biological activities of the hydrolysate are independent of the extent of hydrolysis.

**Table 5:** Regression coefficients for predicting different dependent variables.

Factor	Degree of hydrolysis		DPPH scavenging		ACE inhibition	
	Acid hydrolysis	Enzyme hydrolysis	Acid hydrolysis	Enzyme hydrolysis	Acid hydrolysis	Enzyme hydrolysis
Mean/interaction (β_0_)	−94.330	32.856	26.686	3.805	−3.735	14.109
X1: Temperature (L) (β_i_)	4.057	−0.581	0.741	1.441	0.355	−0.135
Temperature (Q) (β_ii_)	−0.043	0.014	−0.007	−0.006	−0.002	0.003
X2: Time (L) (β_i_)	0.625	−0.083	−0.260	0.149	−0.184	0.074
Time (Q) (β_ii_)	−0.009	0.000	0.006	0.000	0.003	0.000
X3: acid/enzyme concentration (L) (β_i_)	523.542	2.707	398.516	23.125	2,438.897	2.258
Acid/enzyme concentration (Q) (β_ii_)	−8,164.058	0.506	−551.467	−1.576	−27,545.003	−1.148
1L∗2 L (β_ij_)	0.002	0.002	−0.004	−0.004	0.002	0.000
1L∗3 L (β_ij_)	5.944	−0.018	−3.335	−0.218	−7.623	0.177
2L∗3 L (β_ij_)	2.017	−0.009	1.889	−0.060	−0.873	0.001

Abbreviations: ACE, angiotensin-I converting enzyme; DPPH, 1,1-diphenyl-2-picrylhydrazyl.

**Figure 2:**
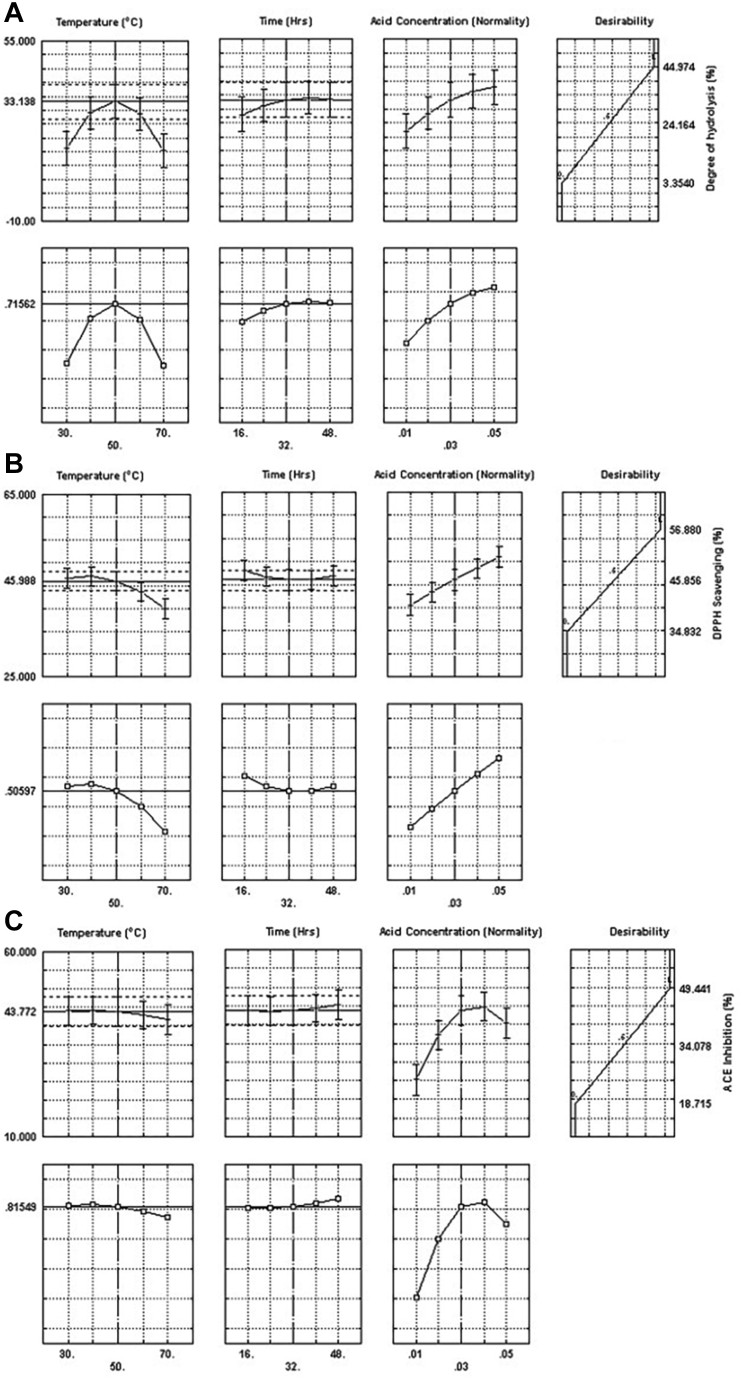
Desirability profile of (A) degree of hydrolysis, (B) DPPH scavenging, and (C) ACE inhibition for acid hydrolysis. Abbreviations: ACE, angiotensin-I converting enzyme; DPPH, 1,1-diphenyl-2-picrylhydrazyl.

The desirability function to obtain a higher value for dependent variables was fitted by the least square method, assigning the dependent variable at the observed low, and high values for the corresponding desirability of 0 and 1, respectively, and the profiles were plotted. These desirability profiles show which levels of the predictor (X1, X2, X3) produce the most desirable predicted response on the dependent variables. The profiles for the predicted response and the desirability levels for DH% (Figure 2A) indicate that hydrolysis conditions of 50°C, 32 h, and 0.03 N HCl result in the optimum DH% of 33.14% with a desirability score of 0.7156, suggesting that an increase in the process variable levels above the optimum levels will not increase the DH% significantly. The desirability profile for DPPH radical scavenging activity indicated that the optimum DPPH radical scavenging activity of 46% could be obtained at the hydrolysis conditions of 50°C, 32 h, and 0.3 N acid concentration (Figure 2B). Above these levels of process variables, the increase in DH% is not significant. The desirability profile (Figure 2C) for ACE inhibitory activity of the hydrolysate indicated that an optimum ACE inhibitory activity of 43.7% could be achieved with hydrolysis conditions of 0.03 N acid concentration, 32 h of hydrolysis at 50°C.

There are very few reports on the chemical hydrolysis of animal blood. Alvarez et al. (2012) reported on the hydrolysis of porcine blood using acids and stated that sulphuric acid and HCl at 6 M concentration could hydrolyze hemoglobin completely. They also observed that increasing the hydrolysis temperature from 50°C to 80°C reduced the hydrolysis time from 20 to 5 h to obtain an average peptide size of 15 to 20 kDa. Alvarez et al. (2013) also attempted alkaline hydrolysis of porcine blood hemoglobin to obtain peptides and amino acids for animal feed. Izgaryshev et al. (2016) obtained DH% of 15 to 20% by hydrolysis of porcine blood with 5% acetic acid or 10% citric acid at 35°C for 12 h. Pakdeeswan et al. (2017) obtained hemoglobin hydrolysate with antibacterial and antioxidant activity by hydrolyzing crocodile blood with 0.05 M HCl at 35°C for 24 h. In the present study, the hydrolysis of chicken red blood cells with 0.03 M HCl at 50°C for 32 h resulted in DH% of 33.14% yielding a hydrolysate with 46% of DPPH radical scavenging activity and 43.7% of ACE inhibitory activity.

### Enzyme Hydrolysis

Alcalase was used to enzymatically hydrolyze chicken red blood cells at different combinations of enzyme level, hydrolysis time, and temperature of hydrolysis and the observed values of DH% and the DPPH scavenging and ACE inhibitory activity of the hydrolysate are shown in Table 3. The experimental data were subjected to ANOVA to understand the effect of main factors and their interactions on the dependent variables (Table 6). During enzymatic hydrolysis of chicken red blood cells, the extent of hydrolysis (DH%) was significantly influenced by the temperature of hydrolysis (*P* < 0.001) and enzyme concentration (*P* < 0.01). However, the time of hydrolysis had no effect (*P* > 0.05) on DH%. Interaction between temperature and time of hydrolysis also had a significant influence on DH%. DPPH radical scavenging of the hydrolysate was marginally influenced by the temperature of hydrolysis (*P* < 0.05), the interaction between temperature and time of hydrolysis (*P* < 0.05), and temperature and enzyme concentration (*P* < 0.05). However, ACE inhibitory activity of the hydrolysate was highly influenced by temperature (*P* < 0.001), enzyme concentration (*P* < 0.001), and their interaction (*P* < 0.001).

**Table 6:** ANOVA for the effect of enzyme hydrolysis conditions and their interactions on degree of hydrolysis, DPPH scavenging, and ACE inhibition of protein hydrolysate.

Factor	Degree of hydrolysis				DPPH scavenging				ACE inhibition			
	Sum of squares	degrees of freedom	Mean sum of squares	F Value	Sum of squares	degrees of freedom	Mean sum of squres	F Value	Sum of squares	degrees of freedom	Mean sum of squares	F value
Temperature (X1)	3,902.45	2	1,951.23	263.69∗∗∗	401.00	2	200.50	4.09∗	872.60	2	436.30	41.68∗∗∗
Time (X2)	38.30	2	19.15	2.59NS	47.58	2	23.79	0.49NS	43.18	2	21.59	2.06NS
Enzyme concentration (X3)	130.67	2	65.33	8.83∗∗	337.35	2	168.67	3.44NS	647.09	2	323.55	30.91∗∗∗
X1∗X2	68.45	1	68.45	9.25∗∗	299.08	1	299.08	6.11∗	0.16	1	0.16	0.02NS
X1∗X3	1.62	1	1.62	0.22NS	228.70	1	228.70	4.67∗	150.22	1	150.22	14.35∗∗∗
X2∗X3	3.53	1	3.53	0.48NS	157.52	1	157.52	3.22NS	0.03	1	0.03	0.00NS
Error	125.79	17	7.40		832.71	17	48.98		177.94	17	10.47	
Total SS	4,270.81	26			2,303.94	26			1,891.23	26		

∗∗∗*P* < 0.001; ∗∗*P* < 0.01; ∗*P* < 0.

Abbreviations: ACE, angiotensin-I converting enzyme; DPPH, 1,1-diphenyl-2-picrylhydrazyl; NS (*P* > 0.05).

**Figure 3:**
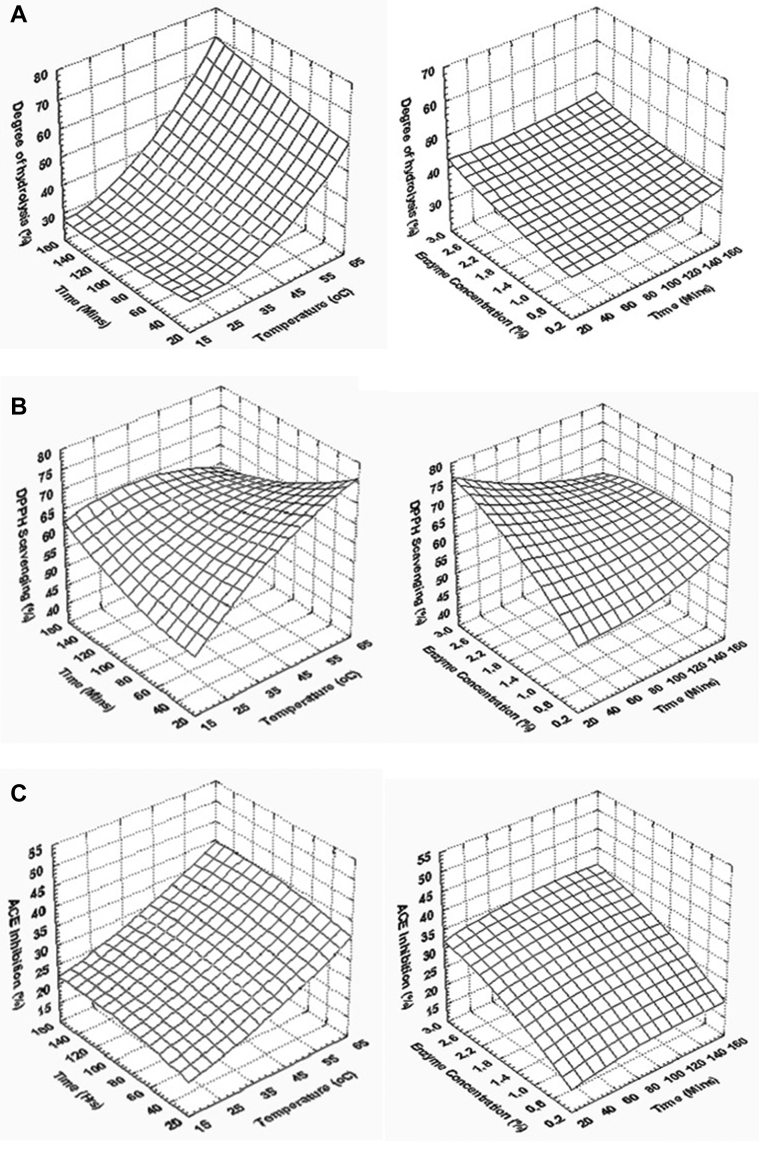
Effect of time and temperature (enzyme concentration 1.5%), (B) time and enzyme concentration (temperature 40°C) on (A) degree of hydrolysis, (B) DPPH scavenging activity of hydrolysate, and (C) ACE inhibitory activity of hydrolysate. Abbreviations: ACE, angiotensin-I converting enzyme; DPPH, 1,1-diphenyl-2-picrylhydrazyl.

The response surface plots show that the DH% increases sharply with increase in the temperature of hydrolysis, whereas increase in the enzyme concentration resulted in a gradual increase in DH%, and no such increase was observed with the increase in temperature of hydrolysis (Figure 3A). DPPH radical scavenging activity as a function of time and temperature of hydrolysis also shows a sharper increase with increase in temperature and gradual increase with increase in time and enzyme concentration (Figure 3B). Even though ANOVA showed no effect of time and enzyme concentration on DPPH radical scavenging activity, this gradual increase in scavenging activity with an increase in time and enzyme concentration might be attributed to the interaction effect with temperature. ACE inhibitory activity showed an increase with increase in temperature of hydrolysis and the level of enzyme used for hydrolysis (Figure 3C).

Regression analysis of the experimental data was carried out to obtain the regression coefficients (Table 5) for the main effects and their interactions to fit a suitable regression equation (Eq.1) to obtain the predicted value of dependent variables. The high *R*^2^ values of 0.9705 and 0.9059 between the observed and predicted values of DH% and ACE inhibition, respectively, indicate that the derived regression equation can be used to determine the value of the dependent variable at different levels of 3 factors, which are influencing them. However, the *R*^2^ value for the DPPH radical scavenging activity was comparatively lower (0.6386). Although no strong correlation was observed between DH% and DPPH radical scavenging activity (*r* = 0.0.38), DH% and ACE inhibitory activity showed comparatively higher (*r* = 0.76) correlation.

**Figure 4:**
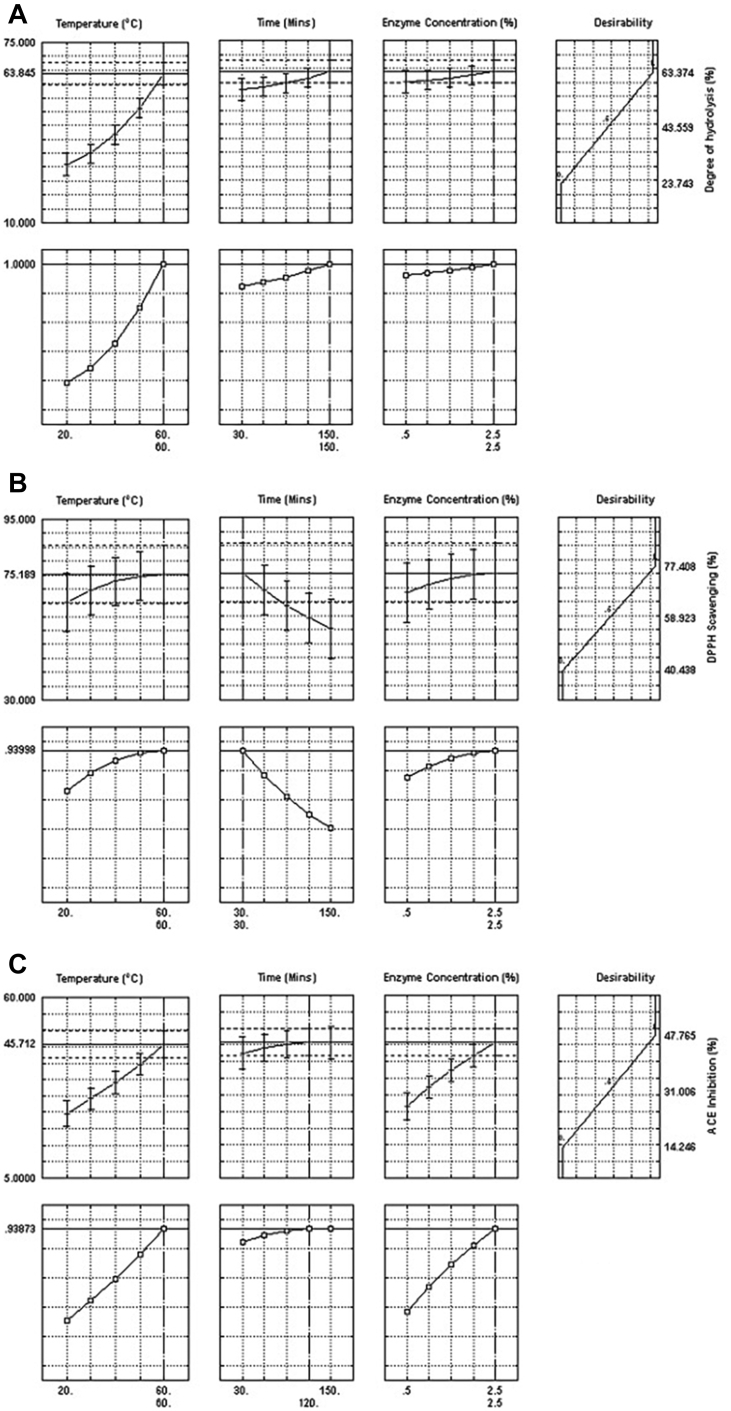
Desirability profile of (A) degree of hydrolysis, (B) DPPH scavenging, and (C) ACE inhibition for enzyme hydrolysis. Abbreviations: ACE, angiotensin-I converting enzyme; DPPH, 1,1-diphenyl-2-picrylhydrazyl.

The profiles for the predicted response and the desirability levels for DH% (Figure 4A) indicate that hydrolysis conditions of 60°C, 150 min, and 2.5% alcalase resulted in maximum DH% of 63.85% with the highest possible desirability score of 1.0. The highest DPPH scavenging activity of 75% was obtained with hydrolysis conditions of 60°C, 30 min, and 2.5% of the enzyme (Figure 4B). The desirability profile (Figure 2C) for ACE inhibitory activity of the hydrolysate indicated that an optimum ACE inhibitory activity of 45% could be achieved with hydrolysis conditions of 2.5% alcalase, 120 min of hydrolysis at 60°C. Similar hydrolysis conditions for higher DH% and ACE inhibitory activity indicate the correlation between the 2, while DPPH radical scavenging activity was higher at lower DH%, which is reflected by the lower correlation coefficient between the 2.

Enzymatic hydrolysis is commonly used to obtain protein hydrolysate having biological activity. Characteristics of the protein hydrolysate depend on the extent of hydrolysis, which in turn is influenced by different process variables such as enzyme concentration, substrate concentration, temperature, reaction time, and pH. Several reports are available on the preparation of protein hydrolysates from animal blood. Perez-Galvez et al. (2011) achieved a maximum DH% (28.89%) of porcine blood by alcalase at pH 6.24, 54.2°C, and enzyme–substrate ratio of 10%. Hydrolysis of porcine blood using a neutral protease at pH 5.0, enzyme to substrate ratio of 0.11, temperature of 45°C, and hydrolysis time of 12 h resulted in a 35.06% DH% (Zheng et al., 2013). Guo et al. (2008) used a mixture of pancreatin and flavourzyme to hydrolyze porcine hemoglobin with 97.69% nitrogen recovery with hydrolysis conditions of 50.4°C, pH 7.8, hydrolysis time of 15.4 h, and enzyme concentration of 0.2%. The use of commercial protease for hydrolysis of duck blood was attempted by Zheng et al. (2014), and the optimal conditions for achieving a high value of trichloroacetic acid solubility index were substrate concentration of 14 g/100 mL, temperature of 51°C, initial pH of 7.0, and time duration of 7.5 h. Huang and Liusd (2010) reported that the DH% during enzymatic hydrolysis of chicken blood meal increases with an increase in hydrolysis time and enzyme concentration. da Silva et al. (2021) used alcalase to hydrolyze chicken blood meal, achieving a DH% of 26% at 50°C, 6.5% enzyme concentration, and pH 8.5. In the present study, chicken red blood cells separated from whole blood were hydrolyzed using alcalase with a maximum DH% of 63.85% under hydrolysis conditions of 60°C, 150 min, and 2.5% of the enzyme. The results of the present study and earlier reports indicate that hydrolysis of animal blood is dependent on blood type, the enzyme used, and the hydrolysis conditions adopted.

**Figure 5:**
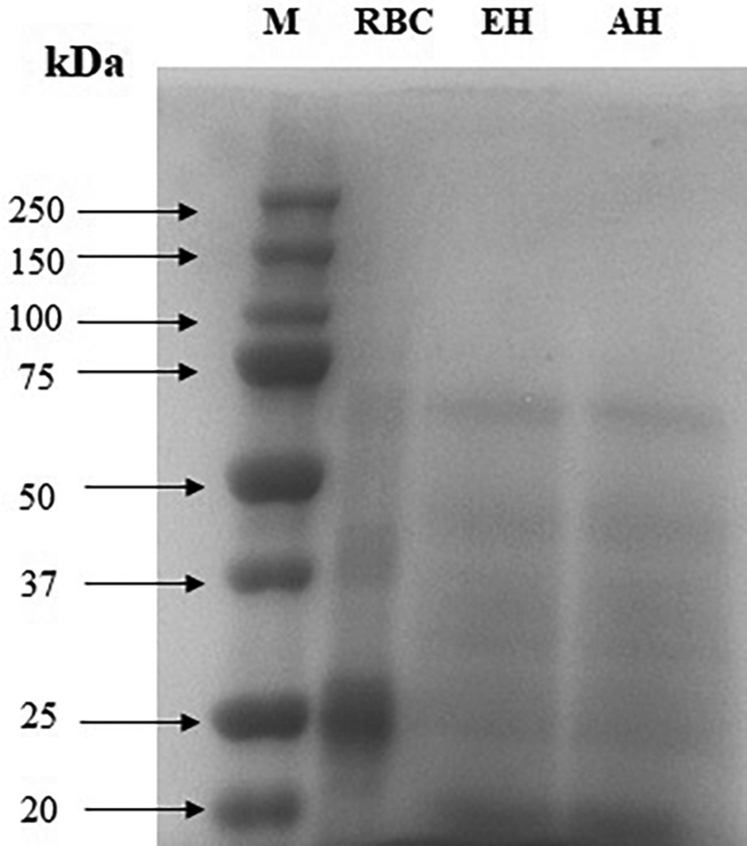
SDS-PAGE electrophoresis of hydrolysate. Abbreviations: AH, acid hydrolysate; EH, enzyme hydrolysate; M, marker; RBC, red blood cells.

The electrophoretic pattern of proteins and fragments in the hydrolysate determined by SDS-PAGE is shown in Figure 5. The red blood cells showed 3 major bands of protein at 25, 37, and 75 kDa. Both the hydrolysates showed 5 electrophoretic bands with molecular weight ranging from >20 to 75 kDa, indicating fragmentation of the protein by hydrolysis. There was no difference in the electrophoretic pattern of acid and enzyme hydrolysates.

The total peptide composition of the protein hydrolysate determines its bioactivity, and the peptide composition depends on the specificity of the enzyme used and the process conditions. In the present study, the effect of the hydrolysis conditions on the DPPH scavenging activity and ACE inhibitory activity of the hydrolysate from chicken red blood cells was evaluated. Protein hydrolysates from porcine plasma (Liu et al., 2009) and bovine plasma (Salgado et al., 2011) have been shown to demonstrate antioxidant activities. Thiansilakul et al. (2007) stated that the type of proteinase, DH%, and defatting process prior to hydrolysis influence the antioxidant activity of the protein hydrolysate. Seo et al. (2015) optimized the conditions for maximum DPPH radical scavenging activity (70.38%) of bovine plasma protein hydrolysate. da Silva et al. (2021) (reported that DPPH scavenging activity of protein hydrolysate from chicken blood meal is not affected by DH%. It was also observed in this study that DPPH radical scavenging activity of the hydrolysate from chicken red blood cells is independent of the DH%, as the hydrolysis conditions for achieving higher DH% and higher DPPH radical scavenging activity were different. However, Liu et al. (2010) reported that the antioxidant activity of porcine plasma protein hydrolysate increased with an increase in DH%.

ACE inhibitory activity of the protein hydrolysate was found to be influenced by the enzyme source and concentration and hydrolysis conditions (Matsui et al., 1999; Kim et al., 2001). There are very few reports on ACE inhibitory activity of hydrolysate from animal blood. Hyun and Shin (2000) developed ACE inhibitory peptides from the alcalase hydrolysis of bovine plasma. Lee and Song (2003) isolated and characterized ACE inhibitory peptides from irradiated bovine plasma protein hydrolysate. Wanasundara et al. (2002) reported that ACE inhibitory activity of bovine plasma protein hydrolysate increases with an increase in DH%, and hydrolysate with 43% DH showed the maximum ACE inhibitory activity. Sampedo and Montoya (2014) found that ACE inhibitory activity of bovine plasma hydrolysate increases with an increase in DH% up to 6.7% and decreases with further increase in DH%. In the present study, the ACE inhibitory activity of the enzyme hydrolysate from chicken red blood cells was found to be influenced by the hydrolysis conditions and was correlated with DH%.

The results of this study indicated during acid hydrolysis of chicken red blood cells, hydrolysis temperature and acid concentration influenced the DH% and DPPH scavenging activity of the hydrolysate while ACE inhibitory activity of the hydrolysate was strongly affected by acid concentration. Acid hydrolysis conditions of 50°C, 32 h, and 0.03 N HCl resulted in optimum DH%, optimum DPPH radical scavenging activity, and optimum ACE inhibitory activity of the hydrolysate. During enzymatic hydrolysis of chicken red blood cells, DH% was influenced by the temperature of hydrolysis and enzyme concentration. DPPH radical scavenging of the hydrolysate was marginally influenced by the temperature of hydrolysis and ACE inhibitory activity of the hydrolysate was highly influenced by temperature and enzyme concentration. Enzyme hydrolysis conditions of 60°C, 150 min, and 2.5% alcalase resulted in the maximum DH%, while the highest DPPH radical scavenging activity of the hydrolysate was obtained under hydrolysis conditions of 60°C, 30 min, and 2.5% of the enzyme. Optimum ACE inhibitory activity of the hydrolysate was achieved at hydrolysis conditions of 2.5% alcalase, 120 min of hydrolysis at 60°C. ACE inhibitory activity of the enzymatically hydrolyzed product was higher at higher DH%, while DPPH radical activity was higher at lower DH%. DPPH scavenging activity of the acid hydrolysate was lower compared to the enzyme hydrolysate, while ACE inhibitory activity of both the hydrolysates was comparable. This study indicated that chicken blood cells could be successfully hydrolyzed by both chemical and enzymatic methods to obtain hydrolysates having antioxidant and ACE inhibitory activity.

## References

[bib1] Alvarez C., Rendueles M., Diaz M. (2012). The yield of peptides and amino acid following acid hydrolysis of hemoglobin from procine blood. Anim. Prod. Sci..

[bib2] Alvarez C., Rendueles M., Diaz M. (2013). Alkaline hydrolysis of porcine blood haemoglobin: applications for peptide and amino acid production. Anim. Prod. Sci..

[bib3] Amarowicz R., Shahidi F. (1997). Antioxidant activity of peptide fractions of capelin protein hydrolysates. Food Chem..

[bib4] Bah C.S.F., Bekhit A.E.D.A., Carne A., McConnell M.A. (2013). Slaughterhouse blood: an emerging source of bioactive compounds. Comp. Rev. Food Sci. Food Saf..

[bib5] Bah C.S.F., Bekhit A.E.D., Carne A., McConnell M.A. (2015). Production of bioactive peptide hydrolysates from deer, sheep and pig plasma using plant and fungal protease preparations. Food Chem..

[bib6] Belhocine D., Mokrane H., Grib H., Lounici H., Pauss A., Mameri N. (2000). Optimization of enzymatic hydrolysis of haemoglobin in a continuous membrane bioreactor. Chem. Eng. J..

[bib7] Clark J.T., Cutler L.J., Meara G.M.O. (1987). Solubilisation of bovine rumen and decolorization of bovine blood by enzymic hydrolysis with alcalase. Meat Sci..

[bib8] da Silva B.A.F.E., Carpin D., Teixiera G.L., Goedert A.C., P Scheer A., Ribani R.H. (2021). Valorization of abundant slaughterhouse byproduct as source of highly technofunctional and antioxidant protein hydrolysate. Waste Biomass Valor..

[bib9] Davila E., Saguer E., Toldra M., Carretero C., Pares D.D. (2007). Surface functional properties of blood plasma protein fractions. Eur. Food Res. Technol..

[bib10] Duan X.J., Zhang W.W., Li X.M., Wang B.G. (2006). Evaluation of antioxidant property of extract and fractions obtained from a red alga, *Polysiphonia urceolata*. Food Chem.

[bib11] Gomez-Juarez C., Castellanos R., Ponce-Noyola T., Calderon-Salinas V., Figueroa J.D. (1999). Protein recovery from slaughterhouse wastes. Biores. Technol..

[bib12] Gomez-Juarez C., Castellanos R., Ponce-Noyola T., Calderon-Salinas V., Figueroa J.D. (1999). Functional properties of globin protein obtained from bovine blood by decolorisation of the red cell fraction. J. Sci. Food Agric..

[bib13] Guo S.G., Zhao M.M., Cui C., Zhang C.H. (2008). Optimized nitrogen recovery and non-bitter hydrolysates from porcine hemoglobin. Food Sci. Technol. Res..

[bib14] Huang S.C., Liu P. (2010). Inhibition of angiotensin I converting enzyme by enzymatic hydrolysate from chicken blood. J. Food Drug Anal..

[bib15] Hyun C.K., Shin H.K. (2000). Utilization of bovine blood plasma proteins for the production of angiotensin I converting enzyme inhibitory peptides. Process. Biochem..

[bib16] Izgaryshev A.V., Babich D.O., Karchin K.V., Bezyukav J.E., Izgaryshev N.V. (2016). Hydrolysis of red blood cells of pig and cattle to ensume optimum conditions for the manufacturing of iron-containing products having maximum heme iron. Bio. Med..

[bib17] Jin S.K., Choi J.S., Yim D.G. (2020). Hydrolysis conditions of porcine blood proteins and antimicrobial effects of their hydrolysates. Food Sci. Anim. Resour..

[bib18] Kim S.K., Byun H.G., Park P.J., Shahidi F. (2001). Angiotensin I converting enzyme inhibitory peptides purified from bovine skin gelatin hydrolysate. J. Agr. Food Chem..

[bib19] Kong B.H., Xiong Y.L. (2006). Antioxidant activity of zein hydrolysates in a liposome system and the possible mode of action. J. Agri. Food Chem..

[bib20] Konieczny P., Uchman W., Krysztofiak K., Przyborski J. (2005). Some selected properties of protein preparations made by enzymatic treatments of animal blood red cell fraction. Acta Sci. Pol..

[bib21] Lee S.H., Song K.B. (2003). Isolation of angiotensin converting enzyme inhibitor peptide from irradiated bovine blood plasma protein hydrolysate. J. Food Sci..

[bib22] Liu Q., Kong B., Jiang L., Cui X., Liu J. (2009). Free radical scavenging activity of porcine plasma protein hydrolysates determined by electron spin resonance spectrometer. Lwt-Food Sci. Technol..

[bib23] Liu X., Song C., Chen R., Jiang X., Jin Y., Zou H. (2010). Identification of angiotensin I-converting enzyme inhibitors in peptides mixture of hydrolyzed red deer plasma with proteomic approach. Chin. J. Chem..

[bib24] Marquez M.C., Vazquez M.A. (1999). Modelling of the enzymatic protein hydrolysis. Process. Biochem..

[bib25] Matsui T., Li C.H., Osajima Y. (1999). Preparation and characterization of novel bioactive peptides responsible for angiotensin I-converting enzyme inhibition from wheat germ. J. Pept. Sci..

[bib26] Pakdeeswan A., Araki T., Daduang S., Payoungkiattikun W., Jangpromma N., Klaynongsruang S. (2017). In vivo wound healing activity of crocodile (*Crocodylus siamensis*) hemoglobin and evaluation of antibacterial and antioxidant properties of hemoglobin and hemoglobin hydrolysate. J. Microbiol. Biotechnol..

[bib27] Perez-Galvez R.M., Almecija C.F., Espejo J., Guadix E.M., Guadix A. (2011). Bi-objective optimisation of the enzymatic hydrolysis of porcine blood protein. Biochem. Eng. J..

[bib28] Ramos-Clamont G., Fernandez-Michel S., Carrillo-Vargas L., Martinez-Calderon E., Vazquez-Moreno L. (2006). Functional properties of protein fractions isolated from porcine blood. J. Food Sci..

[bib29] Salgado P.R., Fernandez G.B., Drago S.R., Mauri A.N. (2011). Addition of bovine plasma hydrolysates improves the antioxidant properties of soybean and sunflower protein-based films. Food Hydrocolloid.

[bib30] Sampedo L.J.G., Montoya J.E.Z. (2014). Effect of hydrolysis and digestion invitro on the activity of bovine plasma hydrolysate as inhibitor of angiotensin I converting enzyme. Braz. Arch. Biol. Technicol..

[bib31] Seo H.W., Jung E.Y., Go G.W., Kim G.D., Joo S.T., Yang H.S. (2015). Optimization of hydrolysis conditions for bovine plasma protein using response surface methodology. Food Chem..

[bib32] Statsoft Inc (1999). STATISTICA for Windows.

[bib33] Thiansilakul Y., Benjakul S., Shahidi F. (2007). Antioxidative activity of protein hydrolysate from round scad muscle using alcalase and flavourzyme. J. Food Biochem..

[bib34] Verma A.K., Kumar C.M., Nitin M., Pavan K. (2017). Efficacy of antioxidant and antimicrobial activity of whole porcine blood hydrolysates and its fractions under *in-vitro* conditions. Anim. Prod. Sci..

[bib35] Wanasundara P.K., Ross A.R., Amarswiz R., Ambrose S.J., Pegg R.B., Shand P.J. (2002). Peptides with angiotensin I converting enzyme (ACE) inhibitory activity obtained from defatted hydrolysed porcine plasma. J. Agri. Food Chem..

[bib36] Wismer-Perdesen J. (1998). Use of hemoglobin on foods – a review. Meat Sci..

[bib37] Wongngam W., Mitani T., Katayama S., Nakamura S., Yongsawatdigul J. (2020). Production and characterization of chicken blood hydrolysate with antihypertensive properties. Poult. Sci..

[bib38] Yang J.H., Lin C.W. (1998). Functional properties of porcine blood globin decolourized by different methods. Int. J. Food Sci. Technol..

[bib39] Yang J., Huang J., Zhu Z., Huang M. (2020). Investigation of optimal conditions for production of antioxidant peptides from duck blood plasma: response surface methodology. Poult. Sci..

[bib40] Yu Y., Hua J., Miyaguchi Y., Bai X., Dua Y., Lin B. (2006). Isolation and characterization of angiotensin I-converting enzyme inhibitory peptides derived from porcine hemoglobin. Peptides.

[bib41] Zheng Y., Chen Q., Shan A., Zhang H. (2013). Optimisation of the enzymatic hydrolysis of blood cells with a neutral protease. Biomed. Res. Int..

[bib42] Zheng Z., Huang Y., Wu R., Zhao L., Wang C., Zhang R R. (2014). Response surface optimization of enzymatic hydrolysis of duck blood corpuscle using commercial proteases. Poult. Sci..

[bib43] Zheng Z., Si D., Ahmad B., Li Z., Zhang R. (2018). A novel antioxidative peptide derived from chicken blood corpuscle hydrolysate. Food Res. Intl..

